# Retraction: One step synthesis of ultrafine PHF@AuNPs nanocomposite and its application in NIR triggered photodynamic therapy

**DOI:** 10.1371/journal.pone.0355452

**Published:** 2026-08-12

**Authors:** The PLOS One Editors

Following publication of this article [1], the Cleveland Clinic Foundation contacted PLOS regarding the provenance and authorization for use of certain published data relating to the XPS results and the PHF-AuNP synthesis methodology presented in [1]. Cleveland Clinic stated that co-author FZ is a former researcher at the Cleveland Clinic, that these data and methodology originated from work performed at Cleveland Clinic and that these were used in [1] without authorization, permission, or appropriate disclosure. Cleveland Clinic further stated that methods covered by US Patent 11,267,708 B2 appear to have informed portions of [1], and that the use of such patent-covered methodology was not appropriately disclosed or authorized.

The authors’ responses did not resolve the above concerns.

PLOS attempted to contact the corresponding authors’ institution Hangzhou City University regarding this matter but did not receive a response.

In light of the concerns pertaining to unauthorized use of patented methodology, and the inclusion of data without permission from the data owners, the *PLOS One* Editors retract this article.

All authors agreed with the retraction.

The retracted article [1] was removed from the *PLOS One* website at the time of retraction. The article’s Copyright and Data Availability statements were also updated at that time, and the removed contents are no longer offered under the Creative Commons Attribution License.
